# Reply: Failing Fontan circulation: Equity in access to heart transplantation via experts in congenital heart disease

**DOI:** 10.1016/j.xjon.2026.102077

**Published:** 2026-08-22

**Authors:** Igor E. Konstantinov

**Affiliations:** Cardiovascular Surgery, Mayo Clinic, Rochester, Minn

Reply to the Editor:

**Figure:**
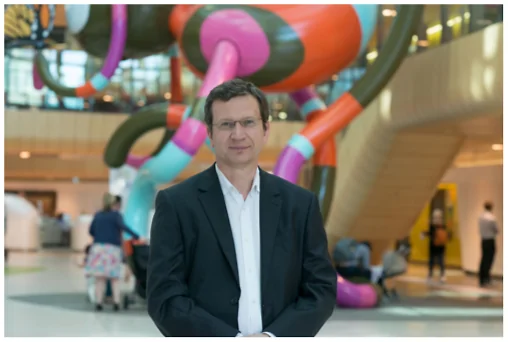

I read the comments of Kearney and colleagues^1^ with interest on the recent article derived from the large cohort of patients of the Australia and New Zealand Fontan Registry.^2^ Putting aside Kearney and colleagues’ speculations on what should or should not be “a marker of success” and “celebration”, their comments address a very important problem that will likely grow to be even more significant during the next decade with an ever-growing population of patients with surgically palliated hypoplastic left heart syndrome.

Needless to say that perceived and real complexities of heart transplantation in failing Fontan circulation, particularly, in patients with hypoplastic left heart syndrome, are numerous.^3^ The patients with failing Fontan circulation may be referred to heart transplantation late or not at all. If the patients with failing Fontan circulation are referred to transplantation to surgeons with limited experience in congenital heart surgery, they may not be accepted because the perceived risk and technical complexity of heart transplantation and the need for extensive vascular reconstruction may be seen as overwhelming.^3^

It was gratifying to see that Kearney and colleagues^1^ quoted our previously published article as “the strongest evidence for access failure” of patients with Fontan circulation to heart transplantation. Indeed, my colleagues and I demonstrated a decade ago that patients with Fontan circulation followed within the national referral centers with expertise in congenital heart surgery were 5 times more likely to undergo heart transplantation^4^ compared with the similar cohort of patients followed outside the centers with a combined congenital heart surgery and transplantation expertise.

It now becomes clear that *all patients* with Fontan circulation must be (1) closely followed by a cardiologist with expertise in congenital heart disease; (2) discussed at the multidisciplinary meeting involving experts in congenital heart disease; and (3) referred for transplantation to surgeons with expertise in congenital heart disease, surgical management of failing univentricular circulation, and heart transplantation after multiple prior heart surgeries. Equity in access to heart transplantation in patients with failing univentricular circulation, relative to potential transplantation candidates without such complex anatomy, can be achieved only if these patients are referred to and have transplantation performed by experts in the surgical management of congenital heart disease.^4^^,^^5^ This approach is best achieved in the tertiary referral centers with expertise in surgical management of failing univentricular circulation.

## Conflict of Interest Statement

Dr Igor Konstantinovis an Associate Editor.

The *Journal* policy requires editors and reviewers to disclose conflicts of interest and to decline handling or reviewing manuscripts for which they may have a conflict of interest. The editors and reviewers of this article have no conflicts of interest.
